# Influence of InAlN Nanospiral Structures on the Behavior of Reflected Light Polarization

**DOI:** 10.3390/nano8030157

**Published:** 2018-03-12

**Authors:** Yu-Hung Kuo, Roger Magnusson, Elena Alexandra Serban, Per Sandström, Lars Hultman, Kenneth Järrendahl, Jens Birch, Ching-Lien Hsiao

**Affiliations:** Thin Film Physics Division, Department of Physics, Chemistry, and Biology (IFM), Linköping University, SE-581 83 Linköping, Sweden; kuoknightly@gmail.com (Y.-H.K.); roger.magnusson@liu.se (R.M.); alese81@ifm.liu.se (E.A.S.); per.o.sandstrom@liu.se (P.S.); larhu@ifm.liu.se (L.H.); kenneth.jarrendahl@liu.se (K.J.); jbh@ifm.liu.se (J.B.)

**Keywords:** InAlN, nanospiral, metamaterial, sputtering, chirality

## Abstract

The influence of structural configurations of indium aluminum nitride (InAlN) nanospirals, grown by reactive magnetron sputter epitaxy, on the transformation of light polarization are investigated in terms of varying structural chirality, growth temperatures, titanium nitride (TiN) seed (buffer) layer thickness, nanospiral thickness, and pitch. The handedness of reflected circularly polarized light in the ultraviolet–visible region corresponding to the chirality of nanospirals is demonstrated. A high degree of circular polarization (P_c_) value of 0.75 is obtained from a sample consisting of 1.2 μm InAlN nanospirals grown at 650 °C. A film-like structure is formed at temperatures lower than 450 °C. At growth temperatures higher than 750 °C, less than 0.1 In-content is incorporated into the InAlN nanospirals. Both cases reveal very low P_c_. A red shift of wavelength at P_c_ peak is found with increasing nanospiral pitch in the range of 200–300 nm. The P_c_ decreases to 0.37 for two-turn nanospirals with total length of 0.7 μm, attributed to insufficient constructive interference. A branch-like structure appears on the surface when the nanospirals are grown longer than 1.2 μm, which yields a low P_c_ around 0.5, caused by the excessive scattering of incident light.

## 1. Introduction

Chirality-induced polarization effect in the cuticle of scarab beetles, such as *Cetonia aurata* and *Chrysina argenteola*, is well-known to reflect light with brilliant color and a high degree of circular polarization [1,2,3]. The reflected circularly polarized light from an incident unpolarized light toward this biological structure is due to its exo-skeleton, which consists of chitin-based layers, each progressively rotated by a small twist angle *θ* with respect to the previous one. Such a naturally helicoidal structure inspires studies of the optical polarization in synthetic chiral nanospirals or a twistedly layer-stacking helicoidal structure for applications such as circular polarizers, bandpass filters, and handedness converters. These are used for, e.g., fiber optical communication, three-dimensional (3D) glasses, and autostereoscopy [4,5,6,7]*.*

Efforts have been devoted to the fabrication and exploration of various chiral structures and materials by methods including glancing angle deposition (GLAD) [8,9], direct laser writing (DLW) [10,11], and holographic lithography (HL) [12]. Different materials can be used to fabricate chiral structures for such polarization-sensitive optical materials, including oxides and fluorides [13,14], gold [11], liquid crystals [15], and silicon nitride [16].

GLAD is the most common technique for the deposition of dielectric chiral materials in the form of either self-assembled or periodically helical columns, including spirals. The mechanism of producing circularly polarized light is described by the interaction between light and the periodic structure, induced by a Bragg phenomenon [17,18]. Accordingly, chirality may be tailored to transform unpolarized incident light to left- or right-handed circularly-polarized light. Large-pitch and long nanostructures with a suite of periods up to several micrometers is required to produce a high degree of circular polarization. This is, however, challenging when using the GLAD technique since broadening and branching of the columns often occur as they grow thicker [13,14].

The DLW method, on the other hand, uses the multi-photon absorption and focused-point laser light with a high magnification objective to write directly into a photoresist. However, at the sharp focal point the intensity of the laser light may lead to local polymerization in areas over one hundred nanometers. This limits the sample size to tens of micrometers, and is thus not economical, both in terms of material usage and process time.

Holographic lithography (HL) is another method, which combining holography and photo-induced polymerization techniques to produce uniform periodic as well as quasi-periodic large 3D structures in photoresist in the optical range [12]. However, this technique is limited to patterning arrayed features or uniformly distributed periodic patterns only. Hence, for the fabrication of arbitrarily shaped patterns, other techniques are required.

Most recently, curved-lattice epitaxial growth (CLEG) [19,20] was demonstrated to produce unique chiral structures by tailoring the nanospiral geometry and internal grading composition of indium aluminum nitride (InAlN) semiconductors. These were demonstrated to have a very high degree of circular polarization (P_c_) in ultraviolet and visible ranges [20,21,22]. A remarkable advantage of CLEG nanorods is their intrinsic curvature, which is fundamentally different from bent rods; bent rods result from a sharp interface between the two different materials with different lattice parameters (such as AlN: 3.11Å and InN: 3.54Å) and coefficients of thermal expansions grown together side-by-side rather than a gradual compositional change. Thus, the latter has a very large internal stress and strain due to lattice mismatch and strained lattices at the interfacial boundary between two materials. In contrast, CLEG curved nanorods have negligible internal stress and strain due to their graded lattice constant and form an epitaxial structure with internal chirality. Furthermore, the lateral compositional gradient can lead to lateral gradients in optical properties. However, detailed information with regard to the effect of CLEG nanospiral structural configurations on the transformation of light polarization is insufficient, which motivates the present study.

The goal of this study is to gain more information about how different growth conditions (nanospiral structural arrangement, shape, geometric confinement, and chemical composition) affect the relationship between the exhibiting circular polarization state and its corresponding wavelength of light reflection. InAlN nanospirals were thus grown by reactive magnetron sputter epitaxy (MSE) with varying external chirality, growth temperatures, TiN seed thickness, nanospiral thickness, and pitch. The optical properties of the resultant nanospirals were analyzed by Mueller matrix spectroscopic ellipsometry (MMSE) and the CompleteEASE software [21,22]. The main P_c_ peak wavelength and band of reflected light were found to be highly correlated with the pitch, length, turn, and composition of the grown InAlN nanospirals. In addition, the possible mechanisms of optical response behavior were discussed.

## 2. Experimental Details

An ultra-high-vacuum (UHV) MSE deposition chamber equipped with two 50 mm-diameter and two 75 mm-diameter targets was utilized to grow InAlN alloys [20,23,24]. The chamber was evacuated to a base pressure of <3 × 10^−9^ Torr with a combination of turbomolecular and mechanical pumps. High-purity 75 mm-diameter Al (99.999%) and 50 mm-diameter In (99.999%) targets were used to co-sputter ternary InAlN under pure nitrogen ambient, supplied as pure nitrogen gas (99.999999%) achieved through a getter purifier. Prior to InAlN growth, a titanium nitride (TiN) seed layer was grown on *c*-plane-oriented sapphire substrates at 850 °C. Typical dc-magnetron powers provided for In, Al, and Ti targets were of 10, 300, and 250 W, respectively. During the sputtering process, a negative substrate bias of 30 V was applied to the sample holder to enhance growth by low-energy ion assistance. Reference [23] A temporal control of substrate rotating angles and the azimuthal orientation of the inclined deposition fluxes were used to tailor the twist-angle *θ* of segments and chirality of the nanospirals, respectively. Here, *θ* = 90° so that four segments completes one turn. More details of CLEG nanospiral growth can be found elsewhere [20].

The composition, crystal structure, and growth plane of the sample surface were determined by *θ*–2*θ* scan X-ray diffraction (XRD) using a Philips PW1820 powder diffractometer with a Cu-K_α_ X-ray lab source. The morphology and surface geometry of the samples were characterized by a LEO-1550 field-emission scanning electron microscope (FE-SEM).

All optical measurements were done in the spectral range of 245–1000 nm at incident angles of 25° using a Mueller matrix spectroscopic ellipsometer (MMSE) produced by J.A. Woollam Co., Inc. (Lincoln, NE, USA) The CompleteEASE software was used to analyze the data measured by MMSE [21,22]. The light polarization state in MMSE measurement was obtained through $S_{o}=MS_{i}$ [11,21], where $S_{o}={\left[S_{o0},\;S_{o1},\;S_{o2},\;S_{o3}\right]}^{T}$ and $S_{i}={\left[S_{i0},\;S_{i1},\;S_{i2},\;S_{i3}\right]}^{T}$ are the polarization states of outgoing and incident lights, respectively, described with the Stokes vector, and **M** is the full 16-element Mueller matrix. For an unpolarized incident light, $S_{i}={\left[1,\;0,\;0,\;0\right]}^{T}$, the outgoing Stokes vector, will be the same as the first column in the normalized Mueller matrix, $S_{o}={\left[1,\;m_{21},\;m_{31},\;m_{41}\right]}^{T}$($m_{11}=1$). An important aspect of the nanospirals in this report is their chirality and potential for transforming unpolarized light into circularly polarized light upon reflection. Thus, the total degree of circular polarization $P_{C}=\frac{S_{3}}{S_{0}}$ of the reflected light is presented.

## 3. Results and Discussion

### 3.1. Nanospiral Chirality

**Figure 1:**
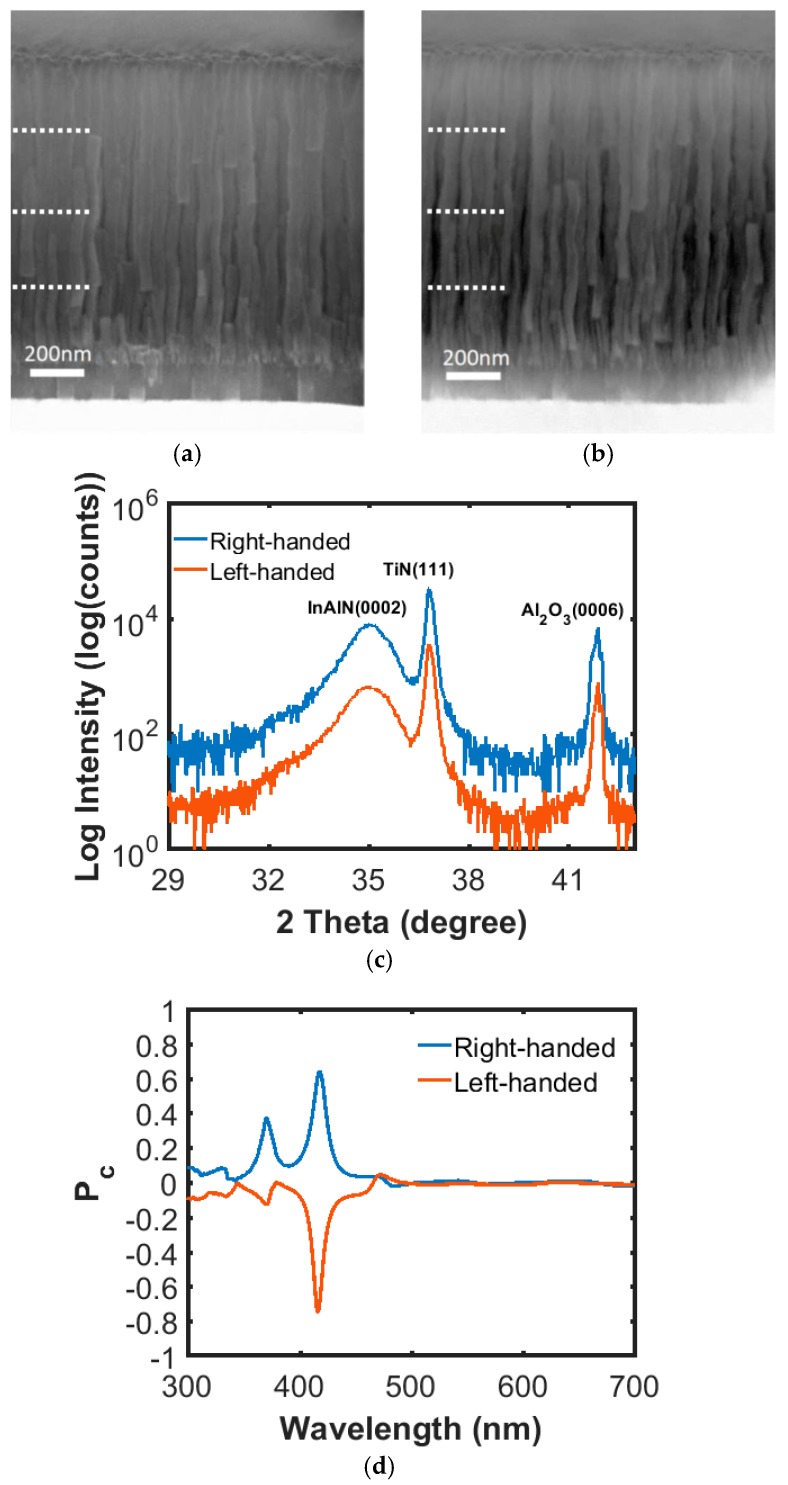
SEM images of (**a**) right-handed and (**b**) left-handed indium aluminum nitride (InAlN) nanospiral film; (**c**) X-ray diffraction (XRD) patterns of right- and left-handed nanospirals; (**d**) P_c_ versus wavelength of the light reflected from nanospirals with opposite handedness. The negative and positive P_c_ is referred to left- and right-handed circularly polarized light, respectively.

Figure 1a,b show side-view SEM images of right- and left-handed InAlN nanospirals, respectively, grown on sapphire substrates assisted with a 120-nm TiN seed layer under identical conditions except for the rotational sense. These chiral nanospirals consisted of four complete turns with a designed pitch of 250 nm, as shown in the images. However, the first pitch is slightly shorter and yields thinner spirals than those in the subsequent pitches, which is attributed to incubation from the self-induced formation of nuclei followed by a coalescence and coarsening process until steady-state growth is achieved in the axial direction [25,26,27]. The crystalline structure of these nanospirals characterized by *θ*/2*θ* scan XRD are shown in Figure 1c. Reflections of InAlN(0002), TiN(111), and Al_2_O_3_(0006) are exclusively present in this scan, located at 35.1, 36.9, and 42.7°, respectively. The SEM and XRD results indicate that these InAlN chiral nanospirals grow preferentially along the *c*-axis and have the same In content of 0.19, as calculated by Vegard’s law without taking strain effect into consideration [23]. The circular polarization measurements of the samples are performed by MMSE. The opposite chirality of the P_c_ curves measured from two samples with different handedness can be clearly seen in Figure 1d. The mirroring shape of the two curves is proof of high reproducibility in the MSE growth process. Though the values of P_c_ are slightly different between the samples; the high P_c_ of around 0.75 at 417 nm indicates that the CLEG InAlN chiral nanospirals are very promising for use as high performance chiral optical elements. It should be noted that the high P_c_ values are an indication that these advanced structures of nanospirals, with complex internal crystal structures instead of homogenous layers [20], cannot be fully modeled using an ideal stratified 90° twisted structure, since that would suggest suppression of the chiral Bragg peaks. While the full modeling of the structures is beyond the scope of the present work, it is the topic of an extensive study, including a larger set of rotation schemes, which is under way.

### 3.2. InAlN Growth Temperature

**Figure 2:**
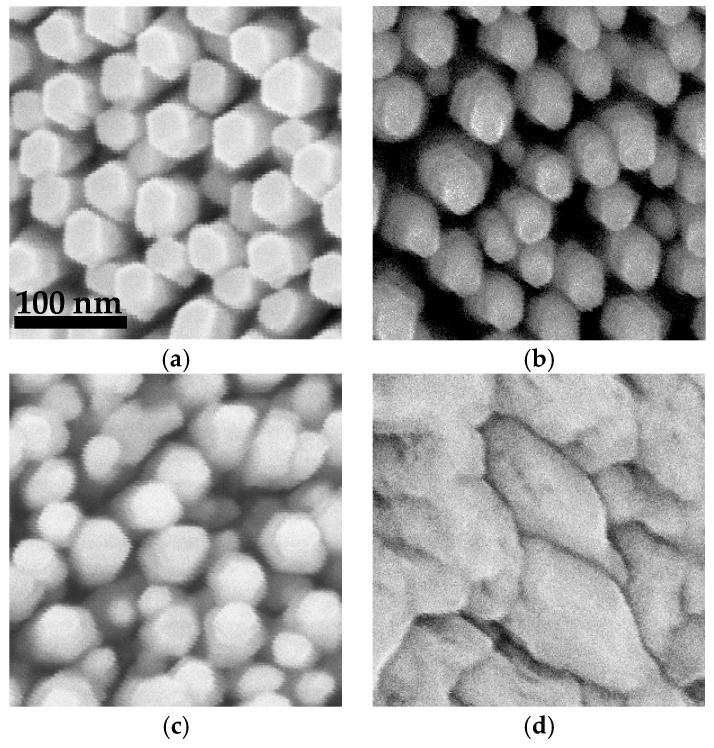
Plan-view SEM images of InAlN nanospirals grown at (**a**) 750 °C; (**b**) 650 °C; (**c**) 550 °C; and (**d**) 450 °C, respectively. All images use the same scale bar as Figure 2a.

Figure 2 shows plan-view SEM images of InAlN grown at temperatures of 450, 550, 650, and 750 °C. Except for the temperature, all samples were grown under the same controlled conditions where left-handed nanospirals where formed by making four complete turns with a transient substrate rotation angle *θ* of 90° between segments. All TiN seed layers had a thickness of 60 nm.

At growth temperatures of 650 and 750 °C, well-separated hexagonal nanospirals are formed, having a well-defined diameter with little deviation as can be seen in Figure 2a,b. When reducing the temperature to 550 °C, the top of the nanospirals becomes tapered with the diameter varying widely from 10 to 50 nm, as shown in Figure 2c. At temperatures lower than 450 °C, the InAlN initially grows with nanospiral geometry (not shown here), but then coalesces, hence forming a thin-solid film-like nanostructure with size bigger than 200 nm, as shown in Figure 2d. In addition, the thickness of the film-like nanostructure is thinner than that of the well-separated nanospirals. Since the volume of the deposited material is the same, a higher growth rate is expected for the well-separated nanostructures. However, a trend of decreasing nanospiral total thickness from 1.19 to 0.85 µm with increasing temperature from 550 to 750 °C is observed, which is attributed to a lower In incorporation rate at a higher growth temperature [26,27].

**Figure 3:**
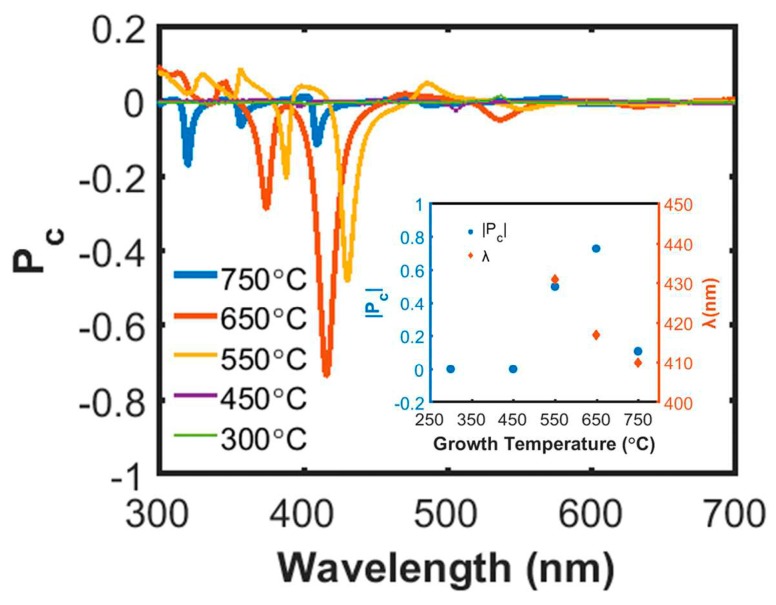
Effect of InAlN nanospiral growth temperature on P_c_ with respect to wavelength. The inset shows |P_c_| and the corresponding peak wavelength versus growth temperature.

The InAlN growth temperature affects not only the morphology, but also the nanospiral material’s optical properties. Figure 3 shows a plot of P_c_ versus wavelength for five different temperatures. Distinct P_c_ peaks can be seen when the growth temperature is in the range of 550–750 °C. The highest |P_c_| of approximately ~0.75 is obtained for the sample grown at 650 °C. |P_c_| decreases to 0.5 for the 550 °C sample, and more dramatically to 0.1 at 750 °C. Almost no P_c_ peak can be identified for the samples grown at temperatures lower than 450 °C. As seen in the SEM images, the sample grown at 450 °C loses the separated nanospiral geometry owing to coalescence, which removes the conditions for constructive Bragg reflection [17,18]. However, the spiral form is retained for the 750 °C sample but its In-content, as determined by Vegard’s law, is found to be around 0.09. This is substantially less than 0.17 ± 0.02 for samples grown at 650 °C or lower, owing to less efficient In incorporation during high temperature growth [26,27,28]. Since lower In incorporation results in a less lateral compositional gradient in the nanospirals, the birefringence contributed from the internal compositional gradient becomes less effective on P_c_ [20,21,22]. This consequence of low P_c_ may indicate that the high |P_c_| obtained from the 650 °C sample is attributed to both external nanospiral geometry and internal compositional gradient [20]. In addition, the wavelength λ for the |P_c_| peak value versus growth temperature is plotted in the inset of Figure 3. The variation of λ shows a trend of shifting towards a shorter wavelength with increasing growth temperature, which can be attributed to a decreased pitch of the nanospirals (due to reduced In incorporation), and is in agreement with the circular Bragg reflection condition λ = $p\bar{n}$, where p is the pitch of nanospirals and $\bar{n}$ is the effective refractive index [17,18].

### 3.3. TiN Seed Layer

**Figure 4:**
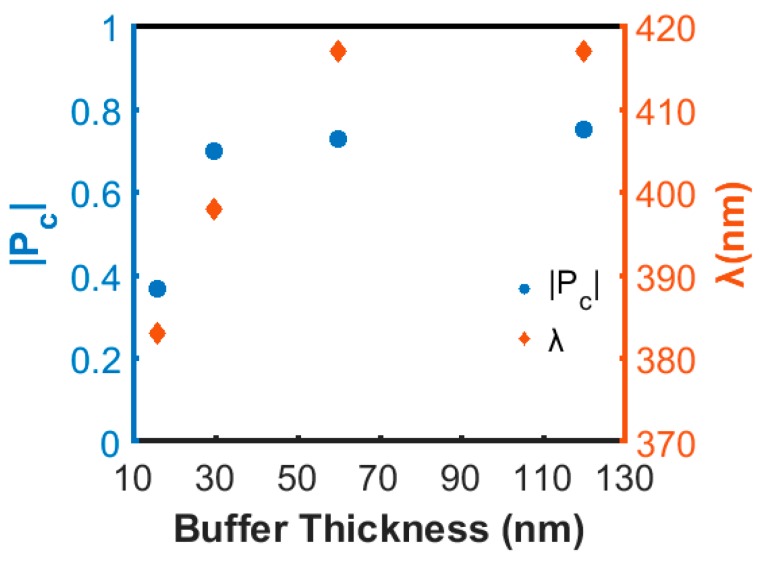
Plot of |P_c_| and corresponding peak wavelength as a function of titanium nitride (TiN) buffer thickness.

In this section, we studied the effect of the TiN seed layer thickness on |P_c_| and the corresponding peak wavelength. The thickness of the seed layer was varied from 16 to 120 nm, while the InAlN nanospiral growth was kept constant using a growth temperature of 650 °C. As can be seen in Figure 4, a large increase of |P_c_| from 0.37 to 0.69 is obtained with increasing TiN thickness from 16 to 30 nm. A further increase of thickness to 120 nm gives slightly increased |P_c_| to 0.75. Also, the peak wavelength λ changes and shifts to longer wavelengths with increasing buffer thickness but remains constant when the buffer is thicker than 60 nm. Since a high |P_c_| results from a strong constructive Bragg reflection, we can speculate that a thicker and thus more reflecting TiN buffer layer minimizes the depolarization and incoherency occurring in a thinner and semi-transparent buffer layer. TiN is reported to be almost opaque for thicknesses larger than 160 nm [29], but already at a thickness of 60 nm most of the light should be reflected from the TiN buffer layer. Together with the observation that the peak wavelength shifts to higher values, we conclude that a larger pitch of well-defined InAlN nanospirals is promoted by the thicker buffer layers.

### 3.4. Total Thickness and Pitch of Nanospirals

**Figure 5:**
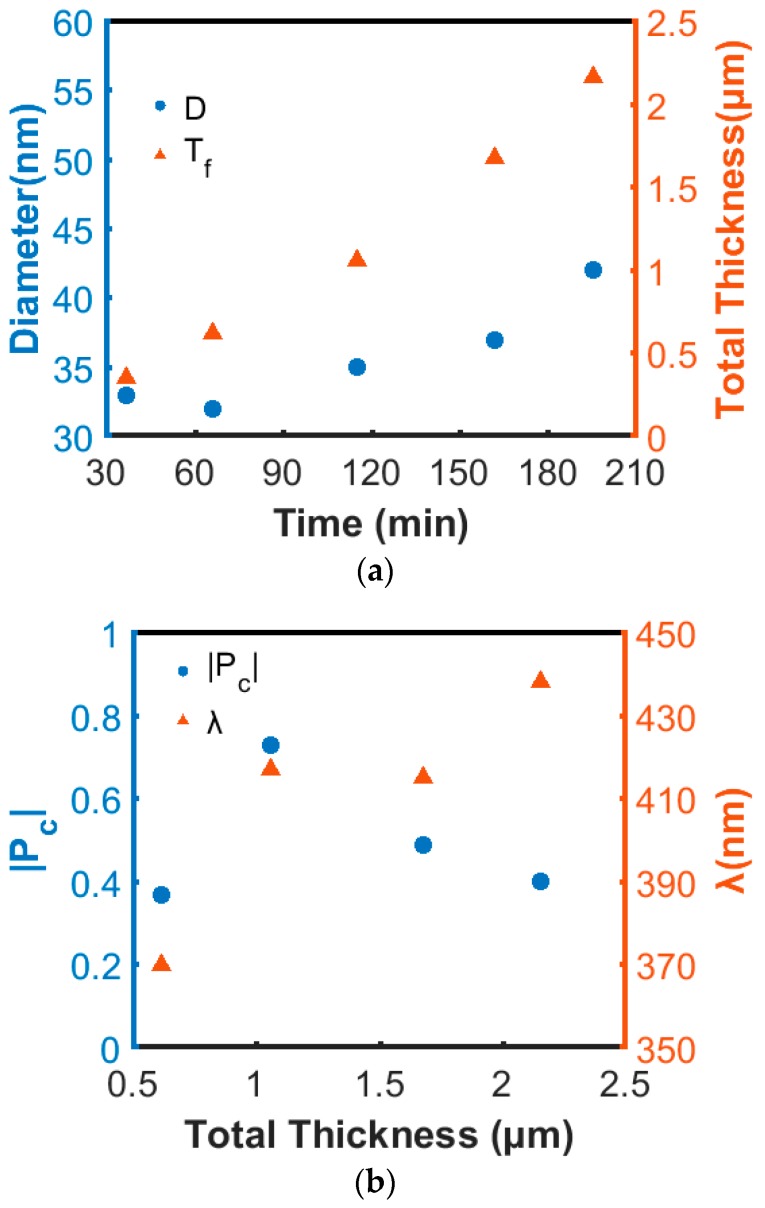
(**a**) Trend of nanospiral diameter (D) and thickness (T_f_) with growth time; (**b**) |P_c_| and λ for different total thicknesses.

This set of nanospirals was deposited on top of 60 nm-thick TiN buffer, with little difference in P_c_ compared to the 120 nm-thick buffer. For the total thickness series, we conducted 1-, 2-, 4-, 6- and 8-turn nanospiral growth experiments, with a designed pitch of 250 nm. All films were formed using a growth temperature of 650 °C. We observed that both the diameter and total thickness of the nanospirals increase with growth time, but the diameter change is less pronounced when the total thickness is smaller than 1 µm, as can be seen in Figure 5a. From side-view SEM images (not shown here), the surface of 6- and 8-turn nanospirals grew rougher and more uneven compared to 4-turn nanospirals, and a branch-like structure was formed for 8-turn nanospirals. The spiral geometry becomes rod-like with some branches owing to the formation of new nucleation sites on the surface. Figure 5b reveals trends in |P_c_| and λ as a function of total thickness. A dramatic increase of |P_c_| from 0 to 0.75 is obtained by adding more turns to the nanospirals, as expected since constructive interference enhances with the number of periods in the structure. However, |P_c_| can be reduced if the periodic structure deteriorates, as suggested by the results obtained from 6- and 8-turn nanopirals. In addition, λ seems to have a small red-shift trend towards longer wavelengths with growth time. Detailed examination of the present nanospiral growth rate indicated an increase over time, with a resulting increase of pitch. Since the sputtering power was fixed for all depositions, we observed an increase of Al cathode voltage correlated with a decrease of current with time. A higher cathode voltage may offer a higher sputtering yield, resulting in a higher growth rate [30].

**Figure 6:**
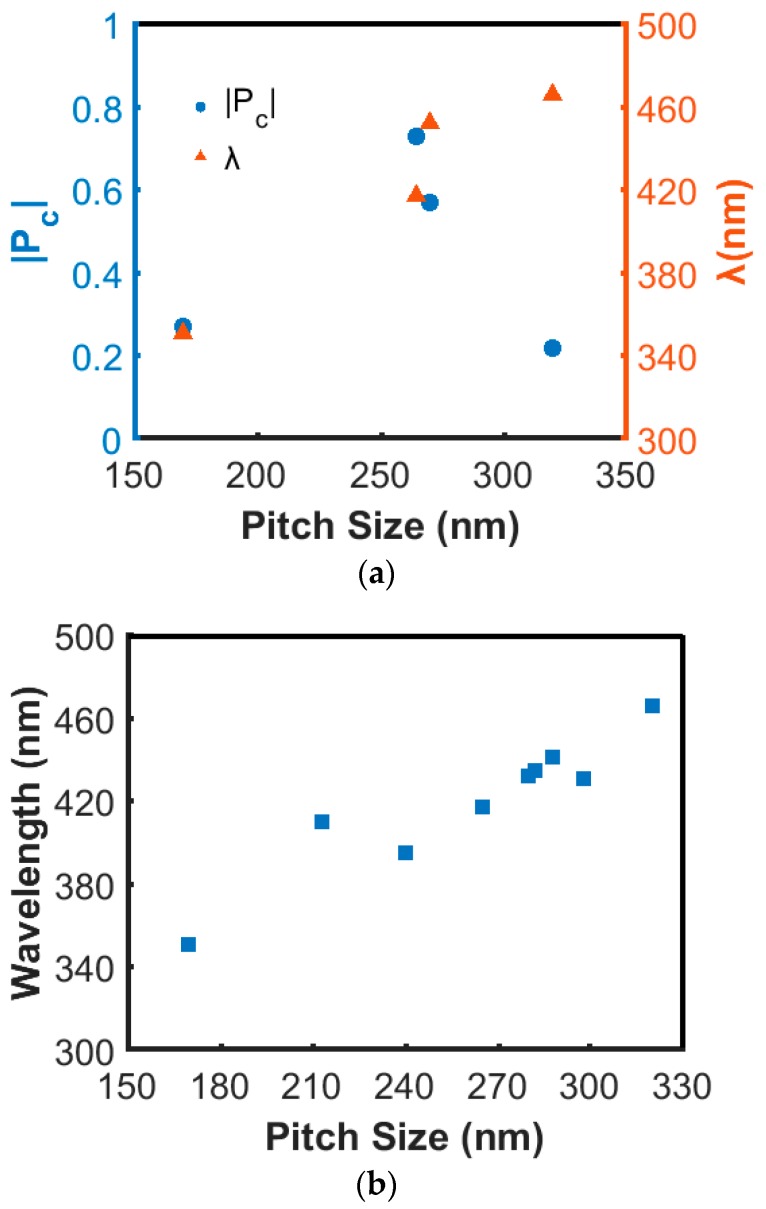
(**a**) The experimental results in P_c_ and λ for different pitch sizes while the total length was kept constant at around 1 um; (**b**) A plot of the relationship between pitch and peak wavelength summarized from all experiments.

Hence, we further studied the effect of pitch on |P_c_| and λ. A series of samples were grown at 650 °C with different pitches, while total thickness was kept constant at around 1 µm. As can be seen in Figure 6a, λ shifts to longer wavelength with increasing pitch. The |P_c_| values are lower for shorter and longer pitch. For shorter pitch, λ is expected to fall into the UV region where the InAlN material has a stronger absorption and thus reduces the reflected light. If the pitch is too long, the film contains fewer periods, leading weaker constructive interference. The peak wavelength position is expected to vary linearly with the pitch according to the Bragg condition. A plot of λ versus pitch analyzed from a larger set of samples, regardless of variation in growth parameters, is shown in Figure 6b. The peak wavelengths in the range of 350–470 nm have high consistency with their corresponding pitch size ranges from 170–320 nm. This monotonic increase of wavelength with pitch is evidence that the circular polarization is connected to the constructive Bragg reflection, as expected. Hence, precise control of the pitch of the grown nanospirals is important for the design of chiral-optical response elements.

## 4. Conclusions

This study reports on the analysis of the polarization behavior of light reflected from InAlN nanospirals grown on *c*-plane Al_2_O_3_ substrates with a TiN buffer layer by reactive MSE with various structural configurations. The degree of circular polarization of the reflected light and the corresponding wavelength range were found to be highly dependent on the buffer thickness, pitch, and morphology of nanospirals. The handedness of the reflected light was also demonstrated to follow the structural chirality. A TiN buffer layer thicker than 30 nm was needed for sufficient reflection. In addition, a film-like structure formed at temperatures lower than 450 °C. When the growth temperature was raised to 750 °C, less than 0.1 In-content incorporated into the InAlN nanospirals. Both cases revealed very low |P_c_| values. A pitch ranging from 200 to 300 nm gave promising |P_c_| higher than 0.5 in the UV to blue region. A transformation of unpolarized to circularly polarized light followed from the circular Bragg condition. Control over the structural configuration and morphology was also demonstrated to be a determinant for obtaining high |P_c_| at a designed peak wavelength.
